# Stem Cell Imaging: Tools to Improve Cell Delivery and Viability

**DOI:** 10.1155/2016/9240652

**Published:** 2016-01-06

**Authors:** Junxin Wang, Jesse V. Jokerst

**Affiliations:** Department of Nanoengineering, University of California, San Diego, 9500 Gilman Drive, La Jolla, CA 92093-0448, USA

## Abstract

Stem cell therapy (SCT) has shown very promising preclinical results in a variety of regenerative medicine applications. Nevertheless, the complete utility of this technology remains unrealized. Imaging is a potent tool used in multiple stages of SCT and this review describes the role that imaging plays in cell harvest, cell purification, and cell implantation, as well as a discussion of how imaging can be used to assess outcome in SCT. We close with some perspective on potential growth in the field.

## 1. Introduction

**Figure 1:**
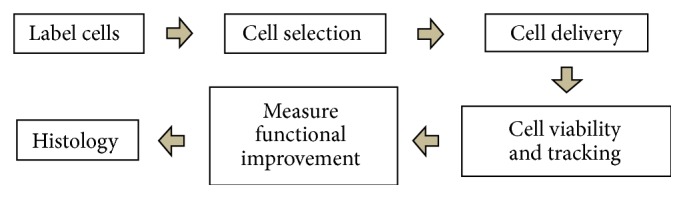
Procedure for SCT. Cells can be labeled with contrast agent either directly or indirectly. The labeled cells are purified from unlabeled cells to obtain a cell product with high signal and thus contrast versus adjacent tissue. Before the delivery, the stability of the labeled cells can be tested to assess any potential toxicity of the imaging agent. After delivery, the viability of the delivered cells is monitored to understand engraftment and survival. The labeled stem cells can be clearly recognized due to increased signal produced by the label. Finally, histology and associated microscopy techniques can confirm that the imaging signal does indeed correspond to the cells of interest.

Stem cells and SCT have remarkable potential in developmental biology, drug discovery, and regenerative medicine [1–6], and imaging techniques are often employed to evaluate the purity, state of differentiation, number, and location of these cells. Stem cells have garnered increasing attention because of their capacity to differentiate into diverse tissue types and increase functional recovery. Stem cell types include embryonic stem cells (ESCs) from the blastocyst, mesenchymal stem cells (MSCs) and bone marrow stem cells (BMSCs) harvested from adults, and induced pluripotent stem cells (iPSC) that are reprogrammed from adult cells via specific transfection factors [7]. Stem cell imaging provides important information about the behavior and function of stem cells including their location, protein expression levels, viability and percent viability, and differentiation status, as well as interactions between the cells and the adjacent tissue [8]. A general outline of stem cell imaging is shown in Figure 1—this in turn is an outline for the rest of this paper. We review the state of the art in functional and anatomic imaging in SCT and regenerative medicine. We highlight the role that imaging plays in stem cell selection and delivery as well as during therapy and for posttreatment validation.

## 2. Stem Cell Preparation

**Figure 2:**
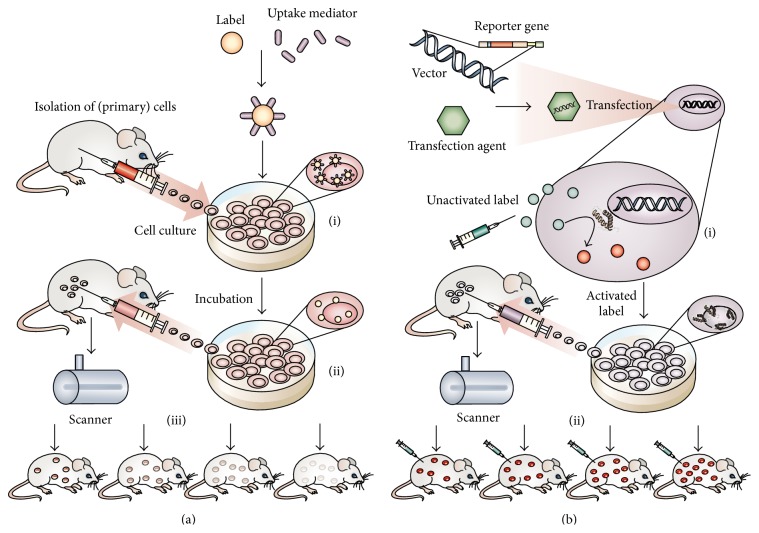
Labeling approaches used in SCT. (a) Direct labeling combines ((a)-(i)) cells and contrast agent and may use a transfection agent to increase the amount of agent that crosses the cell membrane. ((a)-(ii)) The labeled cells are selected from primary cells and are then injected into the target area. ((a)-(iii)) Because the label diffuses as the cells divide, contrast signal will decrease as time passed. (b) In indirect labeling, ((b)-(i)) the cells' genome is modified by reporter gene that encodes for receptors, fluorescent proteins, or enzymes. Except for fluorescent reporter protein, the reporter gene usually does not generate contrast signal itself, but is responsible for the activation of a contrast agent that is added at the time of imaging. ((b)-(ii)) Unlike direct labeling, constitutively expressing genes gene will be copied to daughter cells and the expanded cells can be imaged as well. Reproduced courtesy of Nature Publishing Group [7].

SCT starts with cell preparation, cell labeling, and cell sorting. For example, MSCs must be purified from the bone marrow aspirate, expanded, and labeled. After labeling, cells are sorted to optimize the contrast signal, remove dead or dying cells, and select a population that is positive for the exogenous label or stably expressing the reporter gene. Throughout this section, we will characterize the labeling methods used for cells as either direct or indirect. Most simply, direct imaging uses exogenous labels and indirect imaging transfects cells with reporter genes [9]. The principle and procedure of direct and indirect labeling methods are shown in Figure 2.

### 2.1. Direct versus Indirect Labeling

In direct labeling in SCT, small molecules such as fluorophores, radioisotopes, and nanoparticles are added to the cells during expansion in tissue culture. The labels can be on the cell surface or the cell interior (Figure 2(a)), although confining the labels to intracellular compartments is usually preferred. This is because labels on the exterior could potentially become dislodged and contribute to background signal. Transfection reagents may be used to increase the efficiency of label uptake.

Paramagnetic nanoparticles and lipophilic fluorophores are common contrast agent of direct imaging for magnetic resonance imaging (MRI) and optical imaging, respectively. Probes used for radionuclide imaging include fluorodeoxyglucose (^18^F-FDG) and ^111^In oxine. A complete discussion of all types of direct cell labels is beyond the scope of this paper and the interested reader is referred to reviews dedicated to that topic [10]. The advantages of direct labeling include its simplicity, precise amount of control of label concentration and formulation, and short processing times [8, 9]. However, its applications are limited temporally because the label concentration decreases by roughly one-half in the daughter cells upon every cell division. This decreases the signal as a function of time. Another challenge specific to radionuclide-based imaging is decay of the direct label such as ^111^In oxine [11]. Additionally, direct labeling rarely provides information of the cells' viability and proliferation—that is, the signal is always “on.” This is true even after the cell has died—labels not connected to cells can be misconstrued to be viable cells although direct labels from dead cells are likely scavenging by macrophages or removed via systemic circulation. These disadvantages limit the use of direct labels for the long-term tracking of labeled cells.

Indirect labeling introduces a reporter gene into the genome of the cell of interest to express receptors, enzymes, or fluorescent/bioluminescent proteins suitable for imaging cell location, number, function, and so forth (Figure 2(b)) [12]. These gene products are responsible for either generating contrast signal (e.g., fluorescent green protein for optical imaging) or participating in the reaction with exogenous labels for signal generation (e.g., herpes simplex virus type 1 thymidine kinase (HSV1-tk) for positron emission tomography (PET) or luciferase/luciferin reaction for bioluminescence). Thus, the mechanism of contrast is encoded in the cells' genome with expression being inherited to daughter cells—the amount of signal should be proportional to the cell number. In constitutively expressing cells there should be no decrease in signal intensity between generations [9]. Transient reporter genes may show reduced expression within a single generation or across generations due to silencing of the exogenous reporter gene by the host genome—this is especially common when retroviruses are used to transfect the stem cells [13].

There are also many potential risks that have limited the widespread use of indirect labeling in SCT. Indeed, this approach requires the genome to be altered and often needs viral vectors to facilitate transfection or may need gene silencing [9]. Therefore, indirect imaging is generally only approved in terminally ill patients [14]. Nevertheless, more precise integration of reporter genes into the PPP1R12C locus via zinc finger nucleases shows both high pluripotency and long-term gene expression even in differentiated progeny [15].

### 2.2. Sorting and Purification of Labeled Cells

Neither direct nor indirect SCT labeling can guarantee 100% labeling yield. The labeling efficiency is determined by the surface properties and dose of contrast agents, cell type, membrane coating of the cells, incubation time, and the presence of transfection factor [16–19]. Some direct labels facilitate a calculation of loading efficiency. These cells populations can also be purified to increase the percentage of labeled cells. This is easily done with tags with a fluorescent signal such as dual mode iron oxides or other nanoparticles [18, 20]. Purification is more challenging with radionuclide probes. Fluorescence-activated cell sorting (FACS) is commonly used in sorting cells. This can use the fluorescent signal of an exogenous label or signal from GFP in a reporter gene. Once the equipment recognizes a fluorescent signal, the cells are charged and separated in an external electrical field. Reporter genes such as those transfected by HSV-1tk cannot be easily recognized, and thus a GFP gene is usually added on the same construct as the reporter to guide in FACS sorting [21]. Similarly, cells containing fluorescently labeled magnetic nanoparticles (MRI contrast agent) can be sorted in FACS. Furthermore, magnetically assisted cell sorting (MACS) is an alternative for sorting magnetic particles for MRI [22]. MACS separates magnetic particles in a high gradient magnetic column [23]. However, MACS may also collect magnetic nanoparticles that are not bound to cells—these redundant nanoparticles result in high background.

### 2.3. Characterizing the Labels' Cytotoxicity

The toxicity of contrast agents must be evaluated before using them for SCT. It is generally true that the more concentrated the label is, the higher the contrast signal will be. However, everything, even water, has a toxic dose. An optimal dose of contrast agent should give satisfactory signal and no adverse effects. In the remainder of this section we will describe (1) what factors cause toxicity; (2) how to measure the toxicity; and (3) what the safe dose of contrast agents is.

A label's toxicity depends on various factors including the label material, conjugated ligands or coating, concentration, and the corresponding cell type. One of the main toxic mechanisms of fluorophores is their accumulation in the mitochondria, which disturbs the metabolism of the cell. For example, indocyanine green (ICG) accumulates inside cells resulting in a reduction of the dehydrogenase activity and oxygen consumption in the mitochondria [24, 25]. The toxicity of gold nanoparticles (AuNPs) can be attributed to the formation of reactive oxygen species (ROS) and the intracellular nanoparticle concentration [8]. Cationically functionalized AuNPs cause moderate toxicity, and different ligands cause specific toxicity [26]. Additionally, the remaining surfactant used to grow gold nanorods is also toxic because it is a detergent [27]. In addition, direct labels such as nanoparticles can also perturb downstream cell function. One example of this is altered expression of cytokines in the presence of an exogenous label [28, 29]. A careful and complete analysis of cell toxicity is needed for each novel label type.

Radioisotopes are contrast agents used in nuclear imaging such as single-photon emission computed tomography (SPECT) and PET. Although some radioisotopes and reporter genes for nuclear imaging have been demonstrated to be safe in stem cells [30], using a dosage too high can induce obvious reduction of proliferative activities of stem cells, indicating presence of structural and genetic damage [31]. In addition, excessive auger electrons (a type of electrons emission that occurs when energy between other electron transitions was transferred to the electrons) near the nucleus can cause DNA double bond breaks in stem cells [32]. However, this is relatively rare and is unique to auger emitters for radionuclide therapy [33] and not more typical radionuclides. For MRI, the toxicity of iron oxide nanoparticles is attributed to the generation of ROS [34]. Although the toxicity of the contrast agents varies, it may not always cause cell death. Measuring cell viability is one way to determine the cytotoxicity.

The measurement of cell viability is a straightforward method to determine the cell's behavior and activity as well as to assess the cytotoxicity of external labels. Several toxicity assays based on different mechanisms have been developed to measure the live cell number. Trypan blue exclusion is a cheap method to determine viability and total cell counts of cells. Once the cells are dead, their cell membranes lose the ability to screen uptake from the surrounding environment and become blue [35]. Consequently, live cells only have blue rims while dead cells are stained blue, and the number of live cells can be determined. The MTT assay is another assay that uses 3-(4,5-dimethylthiazol-2-yl)-2,5-diphenyltetrazolium bromide (MTT), a tetrazolium dye, to measure the level of the cell's metabolic activity. MTT can crystalize in the presence of oxidative reduction to give a color change from yellow to blue. The crystals are dissolved in organic solvent and have peak absorption between 570 nm and 590 nm [35]. The absorption is proportional to the cell numbers. Thus, it is a method to compare the viability and proliferation of stem cells with and without contrast agents.

Another method to quantify stem cells' viability is using the resazurin assay. Resazurin is reduced to the fluorescent resorufin by nicotinamide adenine dinucleotide (NADH) or other reductive enzymes. This degree of reduction is proportional to number of viable cells. This assay is highly sensitive and dependent on the cell type, and the viability and proliferation of stem cells can be estimated by measuring their fluorescence [35]. The 2′-7′-dichlorodihydrofluorescein diacetate (DCFH-DA) assay is an intracellular probe for measuring the oxidative stress generated by ROS. Under oxidation, DCFH-DA generates DCF and fluorescence [36]. In other words, in the redox state the amount of ROS can be measured by detecting the intensity of the fluorescence [36]. Alternative schemes such as the detection of lactate dehydrogenase (LDH) are reported [35].

Although the optimal dose for each contrast agent has yet to be determined, some papers have described contrast agent doses that simultaneously have good contrast signal and cause low cytotoxicity. For example, the lipophilic fluorescent tracer DiD was safe to MSCs at 5 pM per cell [37]. Distinct SPECT images were obtained at a concentration of 1.9 pCi/cell even though 0.27 nCi/cell of ^111^In oxine incubated in human MSCs (hMSCs) for 20 minutes and no adverse effects were found in the cells [31]. For radioactive contrast agents, 6 mCi of technetium-99m (^99^mTc)-exametazime (HMPAO) in 10 million stromal vascular fraction cells (SVFCs) showed negligible cytotoxicity and genetic damage for sufficient SPECT/CT signal [38]; ^64^Cu-TETA-CD45 and ^89^Zr-CD45 immunoconjugates have negligible toxic effects on the engrafted human peripheral blood stem cells (hPBSCs) under 40 *μ*Ci/mL [39].

AuNPs are relatively inert but have shown some size-dependent toxicity. In one study, the upper limit dose is 10^12^ particles per mL for 3 to 5 nm AuNPs [40]. Bare superparamagnetic iron oxide nanoparticles (SPIONs) show relatively strong toxicity. For example, a concentration of 2 mg/mL can cause a 60% decrease in the fibroblasts' viability [41]. But the coated SPION can stably exist in cells. Labeling cells with dextran-coated SPION [42], chitosan [43], polyethylene glycol (PEG 2K) [44], and alkyl-polycation [45] at 30 *μ*g/mL, 80 *μ*g/mL, 100 *μ*g/mL iron of SPION, and 7 *μ*g Fe/mL, respectively, shows negligible cytotoxic effects and clear MR signal detection. Ultimately, every label has to be studied because even slight differences can cause major changes in toxicity profiles.

More sophisticated toxicity assays could be performed to analyze characteristics of contrast agents* in vivo* because labeling can influence protein expressions or differentiation potentials of stem cells. For example, MSCs are able to differentiate into osteogenic, adipogenic, and myocardial tissue. Some articles have shown that using silica nanoparticles [29] and PET radioisotopes [46] in MSCs has no obvious influence on differentiation potential and proteomic contents. In MSCs,* fluc-mrfp-ttk* triple fusion gene was found to cause certain degree of alternations in differentiation, but the differentiation potentials were preserved [47]. Similar analyses were also performed in ESCs when FLI/BLI/PET triple fusion genes were applied [30].

After these* in vitro* toxicity assays are performed, the* in vivo* pharmacokinetics, pharmacodynamics, absorption, distribution, metabolism, and excretion can be performed. This is often accompanied by a dose escalation study including an animal study in which a dose that is 100-fold higher than that to be used in humans is given to animals on a weight/weight basis. An excellent overview of the clinical translation process is described in the literature [48].

## 3. Stem Cell Delivery

Delivering stem cells to the target tissue accurately, efficiently, and effectively is a difficult issue that remains to be addressed. Goals include (1) accurate delivery of cells in injured sites, (2) avoiding damaging the host tissue, and (3) maintaining the viability and the proliferation of stem cell after implantation while avoiding the formation of malignancies such as teratoma [49]. Based on the therapeutic application, the delivery approach varies. Here, we introduce some delivery strategies for SCT and some imaging methods that assist the delivery.

### 3.1. Delivery for Cardiac Therapy

**Figure 3:**
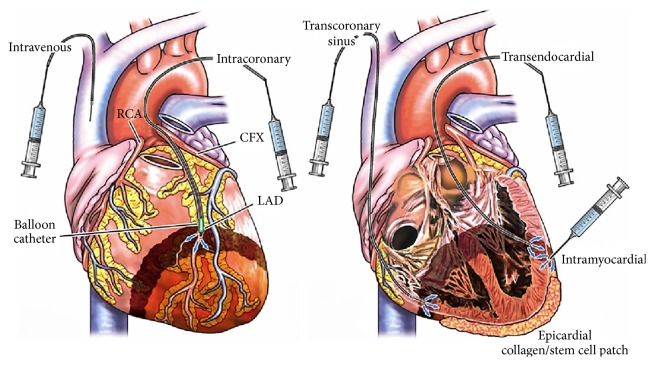
Schematic of stem cell delivery methods for various SCT applications: in cardiac repair, stem cells are either delivered through the vena cava or coronary artery for intracoronary application. Transcoronary sinus or transendocardial or direct injection is used for intramyocardial applications. Reproduced courtesy of Elsevier Publishing group [51].

SCT is a promising treatment for cardiac disease, but tools to more accurately deliver cells to the ischemic region could further improve cardiac function. Approaches to deliver stem cells have been comprehensively reviewed [50]. Although intravenous injection is the easiest approach, it often results in low delivery efficacy—only a small number of cells reach the infarct region [51]. More straightforward alternatives include threading a catheter into the coronary artery (intracoronary transplantation) and direct injection into the cardiac muscle (intramyocardial transplantation) shown in Figure 3 [51]. For intracoronary applications, healthy cells are infused into the infarct zone via a balloon catheter by pressure under percutaneous transluminal coronary angioplasty [51]. One concern about intracoronary transplantation is that implantation into the coronary artery may modulate blood flow in the already damaged area of cardiac tissue. Furthermore, cell clusters or cell fragments could potentially act as microemboli.

For endocardial applications, direct injection into cardiac muscle either through the coronary sinus or coronary artery uses a catheter-based injection system such as the NOGA injection catheter [52]. Transendocardial transplantation requires electromechanical mapping of the heart with a diagnostic catheter and a deflectable tip [53]. After mapping, a small gauge injection catheter and a core lumen transport the cells to the myocardium at a stable slow rate [53]. One limitation of transendocardial transplantation is that it might also cause cell loss in the ventricle and during ventricular arrhythmias [51]. For epicardial intramyocardial applications, stem cells are directly injected to the myocardium outside of the heart [54].

Although there are specific transplantation methods for SCT, misinjection might also be responsible for the poor therapeutic efficacy in SCT cardiac repair. To address this issue, stem cell imaging can provide either static or dynamic images of the implant event. Real-time imaging technologies include ultrasound [55] and photoacoustic imaging [28, 56]. The contrast agent reports the number and location of the stem cell and helps the physician accurately inject the cells in the ischemic region. On the other hand, MRI is commonly used to trace the implanted cells in cardiac infracts, but it can only give information after the surgery.

### 3.2. Delivery for Retinal Therapy

Using stem cells to repair nonfunctioning neuroretinal cells is promising, and the delivery strategies have been improving. Direct injection into the vitreous humor (intravitreal transplantation) and direct injection into the retina (subretinal transplantation) are the main delivery routes for retinal SCT [57]. Alternative injection locations include the optic tract [58] and vein [59]. Compared to subretinal transplantation, intraretinal injection is easier and less invasive and results in higher stem cell survival. However, intravitreal transplantation also suffers from limited efficacy because of the migration barrier between the vitreous cavity and the retina [60]. On the other hand, stem cell transplantation through subretinal injection has better delivery accuracy and higher cell differentiation, but it is technically complicated [61].

Recently, subretinal delivery has been improved with a biodegradable hydrogel-based delivery system that was developed to implant stem cells to the subretina space and results in an even cell distribution and high cell survival rate [62]. The implantation can also be improved via an ultrathin substrate platform which was developed to improve the implantation of retinal pigment epithelium cells in the subretinal space by reducing cell loss [63]. Both approaches increase the safety, viability, and distribution uniformity of subretinal transplantation. Furthermore, in both research and clinical trial, optical coherence tomography has been applied to trace the implanted cells [64] (NCT01773954, the clinical trial registry number on clinicaltrials.gov).

### 3.3. Delivery for Spinal Cord Therapy

SCT is also used in treating spinal cord injury (SCI). Transplantation of stem cells in injured spinal cord can be performed through several routes including systemic delivery [65] and direct intraparenchymal injection [66]. Systematic delivery includes i.v. injection and intrathecal infusion (injection in subarachnoid space). Both transplantation approaches require cells to move to the injury. Intraparenchymal transplantation injects cells directly to the lesion. These three methods have been comparatively studied in the delivery of neural stem/progenitor cells into the injured spinal cord, and it was found that the repeated intralesional transplantation is the most effective and feasible [67]. Similar to the aforementioned therapies, biocompatible scaffolds have assisted with delivery. For example, a fibrin matrix with growth factors can promote the viability and retention of the cells in the lesion site [68].

After transplantation, stem cell imaging is a useful tool to determine the therapeutic efficacy. MRI can be used to monitor the anatomical change of spinal tissue and thus determine the therapeutic efficacy of SCT [69]. Furthermore, because many MRI contrast agents have minimal interfere with the stem cells, MRI can be used to track cells immediately after transplantation [70]. Although reporter genes seem to be the most promising and suitable candidate for long-term monitoring, they are not common for all applications because of the potential risk of gene alteration. On the other hand, because bioluminescent imaging uses indirect labels that are strongly related to the viability and differentiation of the cell, they are powerful tools for studying the efficacy and safety of different stem cells in treating spinal cord injury in small animal models [67] including schemes to evaluate the extent of tumorigenesis.

Delivery methods of stem cells for treating other organs or tissues have been widely studied. For instance, stem cell delivery for knee cartilage repair are either under direct injection or arthroscopic surgery with or without additional scaffolds, growth factors, platelet rich plasma, and gene therapy [71]. Similarly, direct injection of stem cells and placing natural or synthetic matrix/scaffolds that contain stem cells into the periodontium are two main delivery approaches for periodontal tissue regeneration [72]. For facial nerve regeneration, stem cells are first prepared into biocompatible tubes made of poly(lactic-co-glycolic acid) (PLGA) or conduits made of chitosan. The tubes and conduits are later implanted at the injury transection [73]. Nevertheless, the ideal delivery strategies for various SCT applications have yet to be determined. Ongoing work will develop delivery methods that are less invasive with more accurate and efficient fusion to the damaged tissue with minimal cell death.

## 4. Imaging Modalities and Their Contrast Agents

### 4.1. Magnetic Resonance Imaging

MRI can provide whole body, high resolution images by measuring changes of magnetic field with excellent soft tissue contrast. Spinning charged nuclei such as hydrogen atoms that have unpaired protons and neutrons generate magnetic moments that can be aligned parallel or antiparallel to the longitudinal axes when an external magnetic field is applied [74]. Subsequently, while the primary magnetic field remains a radiofrequency (RF) pulse is applied transversely to align atoms at the appointed location to the transverse axes. Once the RF energy is retrieved, the nucleus relaxes back to longitudinal axes at different speeds due to interactions with environment known as the spin-lattice relaxation. The relaxation time is T1. Meanwhile, the exchange of energy between nuceli disturbs the coherence of procession. The nucleus gradually processes at different phases at the transverse plane, where the time for complete out-of-phase relaxation is known as the spin-spin relaxation time, T2. Different tissues or organs have various relaxation times and therefore by measuring the longitudinal and transverse magnetic field, respectively, the types of tissue are determined [12]. Unlike conventional imaging modalities, MRI is more favorable because it uses magnetic field instead of the ionizing radiation. Furthermore, it has unlimited depth of penetration because of the low attenuation of the magnetic field in tissue. On the other hand, it does not show functional, metabolic, and molecular information. It is also time consuming and costly, which limits its wide use in clinics and fundamental research.

MRI also is largely limited in its ability to retrieve functional, metabolic, or molecular information from imaging datasets. However, next generation genes are making progress in this field [75]. For example, the tyrosinase gene can promote the production of melanin that can chelate metal ions [76]. The* mms6* bacterial gene is able to uptake intracellular iron and form cluster of nanoparticle within and outside the cell membrane [77]. A replication-defective adenovirus encoding metalloproteins from the ferritin family reporter is capable of sequestering endogenous iron from the organism [75]. Other MRI reporter genes for cell-based cancer therapies have been studied and reviewed [78]. However, these reporter genes have not been fully evaluated in clinical SCT [74]. Overall, the presence of contrast agent makes cellular detection possible for MRI and makes it desirable in tracking grafted stem cells in SCT.

**Figure 4:**
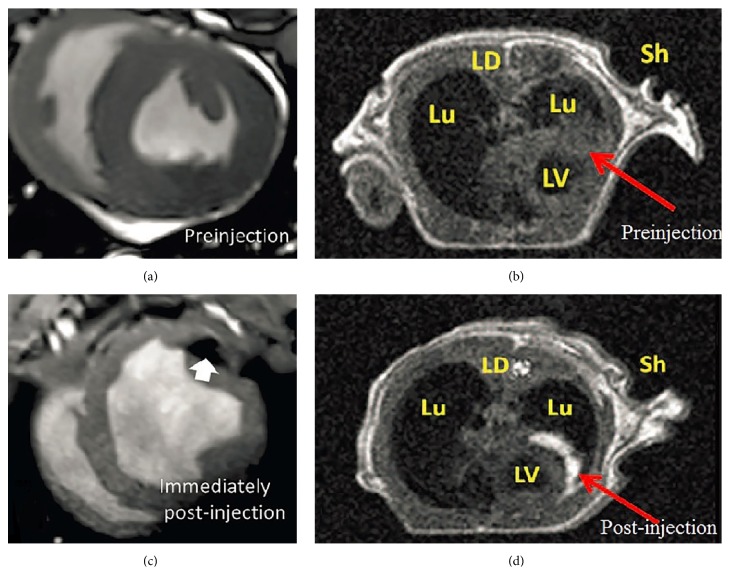
MR image of contrast agent labeled stem cells in preclinical models. (a), (b) are images taken before the therapy and (c), (d) are images taken after the injection of stem cells. Iron oxide nanoparticles were labeled to MSCs and highlighted the cells as darker color pointed by the arrow in image (c) while Gd^3+^ nanoparticle highlighted the cells in bright color as shown by the arrow in image (d). LV: left ventricle; Lu: Lung; LD: lattisimus dorsi; Sh: Shoulder. (a), (c) are reprinted with permission of Wolters Kluwer Health, Inc. [87] and (b), (d) are reprinted with permission of the American Association for the Advancement of Science [29].

In SCT, appropriate contrast agents help distinguish and trace transplanted cells in the lesion after the therapy. There are two types of MRI contrast agent that can strongly influence T1 relaxation time and T2 relaxation time. The T1 relaxation time depends on the dipole-dipole interaction between contrast agent and its surrounding environment. T1 contrast agents shorten T1 rather than T2. For example, clinical T1 contrast agents often contain gadolinium (Gd^3+^) that has strong paramagnetism because of its seven unpaired electrons. The relaxation time of water molecule is shortened when they interact with these unpaired electrons, which makes the MR signal more intense (Figure 4(d)). These ion metals can cause cytotoxicity and are usually packaged with chelates complexes such as 1,4, 7,10-tetraazacyclododecane-1,4,7,10-tetraacetic acid (DOTA) [74] or diethylene-triamine penta-acetic acid (DTPA) [79]. FDA-approved T1 contrast agents used to image stem cell include Gd-DTPA (Magnevist) [80], Gd chelate gadodiamide (Omniscan) [81], and Dg-HPDO3A (Prohance) [29]. Labeling stem cells may or may not need transfection agents [29]. In addition to the relaxation effect, there is also a magnetic susceptibility effect.

T2 contrast agents (or negative contrast agent) act as a small magnet and affect T2 relaxation time by introducing magnetic fields to tissues through water diffusion. Ferromagnetic and superparamagnetic iron oxide nanoparticle (SPION) are typical T2 contrast agents used in the clinic [29]. In SCT, SPION-labeled stem cells are darker in organs such as kidney or lymphoid tissues that have intense signal in MRI [29] (Figure 4(c)). SPIONs have been reported in some preclinical studies such as cardiac repair [82] and knee joint repair [83]. Typical commercial SPION contrast agents include dextran-coated SPION Feridex and carboxydextran-coated ultrasmall SPION Revosit. These require transfection agents to be integrated into the cells and these agents have some toxicity [84]. To address this issue, new SPION contrast agents such as citrate-SPION have been reported to label stem cell without transfection agent [84, 85]. Furthermore, a recent report showed that i.v. injected SPIONs can accumulate in the bone marrow and label stem cells* in vivo* because of the bone marrow's role in the reticuloendothelial system—this is an important and novel approach to labeling stem cells without transfection agents [86].

### 4.2. Nuclear Imaging

Nuclear imaging of SCT can use PET or SPECT. PET tracers can be produced in a cyclotron. The mechanisms of contrast are based on emission of a positron by the PET reporter. These positrons travel through the local environment where they finally lose kinetic energies and interact with electrons—this interaction is called annihilation. The change in energy during the annihilation energizes two 511 keV photons roughly 180° from each other [12]. A gamma ray detector ring, or a set of rings, captures these photons at 360 degrees and converts electrical or optical signal from a scintillator to sinogram which is further reconstructed for tomographic images [12]. Well-known radionuclides include ^18^F, ^64^Cu, ^111^In, and ^68^Ga [74]. SPECT tracers produce only one photon, which is generated from decay of the tracer.

**Figure 5:**
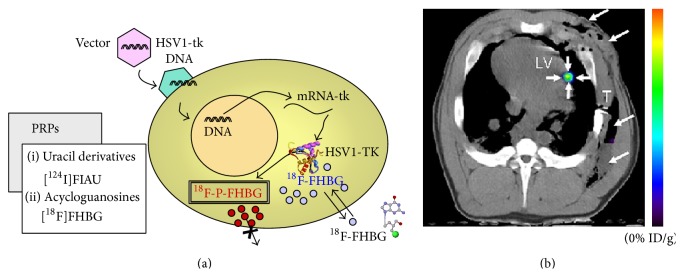
PET images of contrast agent-labeled stem cell. Panel (a) is the schematic of the indirect labeling using HSV1-TK and radioisotopes. Cells are transfected with HSV1-tk via vectors. ^18^F-FHBG is phosphorylated by the enzyme which is encoded in the gene of the cell, and the reporter is consequently entrapped in the cell. Reproduced courtesy of Springer Publisher Group [95]. Panel (b) is a PET-CT image of MSCs that were labeled with a mutant HSV1-tk reporter gene. The cells were implanted into a swine and subsequently administrated with [^18^F] FHBG. The PET-CT image provides both anatomical information (gray scale from CT) and the contrast of the implanted cells (color from PET data). Reproduced courtesy of Radiological Society of North America [96].

The most common PET reporter is a small molecule with high avidity for the glucose-transporter known as ^18^F-FDG. ^18^F-FDG is most commonly used in cancer imaging, but it has also been shown to image stem cells [88]. However, reporter genes are more common for PET imaging in SCT. HSV1-tk and its mutants are typical group PET reporter genes that specifically phosphorylate radioisotopes such as 9-(4-^18^F-Fluoro-3-[hydroxymethyl]butyl)guanine (^18^F-FHBG) [89] (Figure 5(a)), 2′-^18^F-fluoro-5-ethyl-1-beta-D-arabinofuranosyluracil (^18^F-FEAU) [90], and ^131^I-2′-fluoro-2′-deoxy-1-beta-D-arabinofuranosyl-5-iodouracil (^131^I-FIAU) [91]. The phosphorylated radioisotopes get entrapped in the cell.

A protocol of using HSV1-tk and its mutants in tandem with ^18^F-FHBG (FDA approved) has been reported for cancer imaging [92], and its applications in SCT were reported as well [14]. Unlike direct labeling with radionuclides such as ^111^In oxine, the use of HSV1-tk can use a fresh injection of reporter for each imaging event. This overcomes some limitations related to radionuclide decay that hampers direct imaging with PET.

Overall, PET has advantages but also suffers some limitations. PET has high sensitivity at limitless depth of penetration, quantitation capabilities [12], and the ability to image cell viability via a reporter gene. Importantly, human reporter genes such as human mitochondrial thymidine kinase type 2 (hTK2) [93] and human somatostatin receptor 2 (hSSTR2) [94] showed potential to be used in human without inducing immune response. Furthermore, PET can provide earlier diagnostics than anatomical technique such as CT and MRI because it detects biochemical changes which generally occur before anatomical changes in disease [12]. While PET imaging does not provide anatomical information, it is often combined with CT (Figure 5(b)) or MRI to locate the transplanted cells and understand their location relative to adjacent anatomy.

SPECT is another nuclear imaging technique for stem cell tracking, but it uses different radioisotopes and setup. SPECT uses heavy radioisotopes including ^99^mTC, ^123^I, and ^111^In. SPECT radioisotopes can decay by electron capture. The proton in unstable nucleus combines an electron from the inner shell to form a neutron and emits an electron neutrino, where simultaneously auger electrons and gamma ray photons are generated when electrons at outer shell fill up the inner shell. Generally, SPECT radioisotopes have a comparably longer half-life than PET radioisotopes, and thus they are more favorable to those facilities that have limited ability of on-site cyclotron [97]. They emit photons at different energies at one angle when the excess protons decay. To increase the spatial resolution collimators made of tungsten or lead are placed in front of the gamma camera. But this also causes low detection efficiency of the photon, which results in a low sensitivity and a small field of view. Sodium iodide symporter (NIS) is one of the most widely studied reporter genes for SPECT, and ^123^I is used with NIS for stem cell long-term tracking [98–100]. On the other hand, in direct labeling, the stem cells can be labeled with the radioisotopes compounds [101] through incubation for short-term monitoring of grafted cells. Similar to PET, SPECT has high sensitivity but lacks anatomical frames and needs to “cooperate” with other modalities such as CT and MRI.

### 4.3. Optical Imaging

Optical imaging is a relatively cheap and easy technique that records the fluorescent or bioluminescent signal from samples. Typical technologies are fluorescence imaging and bioluminescent imaging. Fluorescence requires an optical excitation source—usually a filtered narrow band light—to excite the contrast agents and a cooled charged couple device (CCD) camera to receive the emission photons. Fluorescent contrast agents include fluorescent protein (e.g., GFP) [102], organic fluorophores [103, 104] (e.g., indocarbocyanine dye), and quantum dots [105–107] (e.g., semiconductor nanoparticles). Fluorescent proteins are indirect labels and fluorophores and quantum dots are direct labels. Quantum dots have advantages over organic fluorescent dyes because of their narrow, symmetric emission, broad excitation, high quantum yield, high molar extinction coefficient, and exceptional resistance to photo and chemical degradation [108].

Fluorescent dyes and quantum dots can also be directly labeled inside or on the cell membrane via peptides (Qtracker cell labeling kit) or lipid (NeuroTrace lipid tracers). Fluorescent imaging has been widely applied in bioimaging research because of its relatively low cost, capability of multiplexed imaging, and good temporal resolution [12]. These properties are favorable for tracing stem cells but can also lead to toxicity (cadmium quantum dots) and autofluorescence [12]. The main challenge in fluorescent imaging is the low depth of penetration caused by the scattering and diffusion of soft tissues. In larger experiment subjects (larger than mice), the optical information strongly attenuates in the body and becomes too weak to be detected before it reaches the detector. Using NIR fluorescent proteins or fluorophores can alleviate this issue, but it only increases the optical penetration by millimeters. Therefore, in SCT, fluorescent imaging is mostly applied in cellular studies, small animal research, and histology rather than human clinical trials. Work in the second NIR optical window is making inroad on this challenging issue [109].

**Figure 6:**
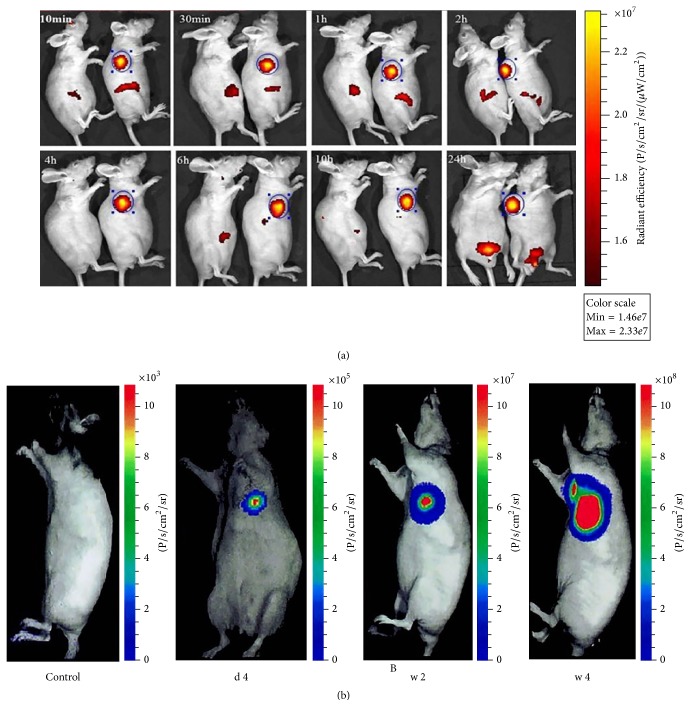
Optical imaging of transplanted stem cells. (a)* In vivo* fluorescent images of gastric cancer mouse 10 min, 30 min, 1 h, 2 h, 4 h, 6 h, 10 h, and 24 h after injection of DiR labeled murine ESCs. Reproduced courtesy of the Ivyspring International Publisher Group [104]. (b) Bioluminescent image of implanted ESCs in weeks 1, 2, and 4. (b) shows that the luciferase reporter gene is capable of performing long-term tracking in the migration and viability of implanted cells, which in this case form a teratoma. Reproduced courtesy of Wolters Kluwer Health, Inc. [114].

Bioluminescence is an indirect, optical imaging technique that uses reporter gene to express specific proteins. The protein (enzyme) reacts with extraneous substrates and generates photons inside the cell without excitation. A well-known example is the firefly luciferase that emits photons by oxidizing D-luciferin in the presence of oxygen, ATP, and magnesium. Several other luciferase enzymes include sea pansy luciferase, Renilla reniformis (RLuc), and marine cope pod luciferase,* Gaussia princeps* (Gluc) [12, 110–113]. The only setup for bioluminescent imaging is a sensitive camera (cooled CCD). Similar to fluorescent imaging, the application of bioluminescence is subjected to the low transmission of light in body, which limits bioluminescence in the studies of small animal (Figure 6(b)) and cell culture [74], but bioluminescence does have much lower background signal than fluorescence and thus is more sensitive.

### 4.4. Ultrasound Imaging

Ultrasound imaging is not used as commonly as the other modalities, but it has significant potential as a real-time, high resolution imaging technique to guide stem cell delivery. Because sound waves propagate at different speeds in different tissues, contrast is formed based on the acoustic impedance at the interface between two tissues [12]. Ultrasound imaging uses a transducer to send ultrasonic waves and receive echoes produced by the interface [12]. Images are reconstructed by analyzing the amplitude, frequency, and reflection time interval of the reflected signal [12]. In cellular imaging, ultrasound imaging is limited to the poor contrast. To address this issue, several types of contrast agents have been developed including microbubbles [115], silica [116], and gold nanoparticles [56]. Microbubbles can stably label and highlight cells for minutes after the delivery [117]. Microbubbles can be controlled and deliver the cells to the targeted area [118], but due to their micron size microbubbles are limited to image the cell surface—thus, they can easily become dissociated from the cells and result in aberrant signaling.

**Figure 7:**
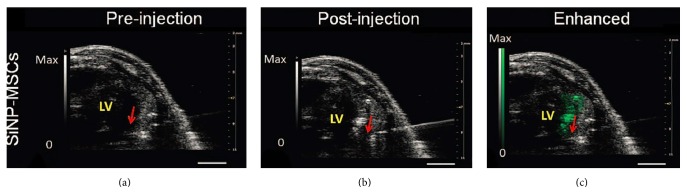
Ultrasound imaging of SCT. Silicon nanoparticles (SiNP) enhance the ultrasound signal of MSCs after cardiac implantation. Ultrasound resolves the needle catheter and the tissue. As shown in (c), the contrast signal due to the backscatter of SiNP highlights the implanted cells. Green signal indicates presence of stem cells. Red arrow highlights the bevel of the needle. Reproduced courtesy of the American Association for the Advancement of Science [29].

Nanosize particles made of silica [119] or mesoporous silica [116] have been developed to overcome this limit and assist with imaging stem cells in SCT. Ultrasound-guided transplantation can improve the accuracy of the stem cell delivery (Figure 7). Ultrasound imaging of stem cells via reporter genes is quite rare, but genes that produce a cell surface protein to be imaged with targeted microbubbles has made this possible [115]. Overall, ultrasound imaging is a desirable imaging modality due to its advantages such as relatively inexpensive, high spatial, and temporal resolution, relatively large depth of penetration, using safe mechanical waves, and high sensitivity [12]. But it also has limitations such as a high background signal and poor specificity. Nevertheless, the ability to obtain anatomical information and definitive molecular information about the cells makes it a promising technique.

### 4.5. Emerging Imaging Modalities

In addition to the aforementioned modalities, there are several modalities gaining popularities in the research of regenerative medicines. Computed tomography is a clinical imaging technique that generates three-dimensional images. Regular organs and tissue are not sensitive to X-ray due to low absorption, but CT can image stem cells that are labeled with metal contrast agents. For example, AuNPs coated with glucose have been used to label MSCs that were imaged with CT after brain implantation [120]. Ultrashort single-walled carbon nanotubes containing bismuth (bioCl/Bi_2_O_3_) have been used to label pig MSCs for CT imaging [121]. SPION-labeled (EndoremTM Iron Oxide NPs) CD133^+^ stem cells in muscle biopsies show contrast signal under CT [122]. The primary challenge with CT-based functional imaging is the low sensitivity of the technique. Very high cells counts are needed for imaging with CT.

Photoacoustic imaging (PAI) compensates for the poor contrast of ultrasound. The configuration includes a laser that is able to selectively excite the contrast agent previously labeled to the stem cell. The heated contrast agent causes thermoelastic expansion and generates acoustic signal. For example, gold nanorods can absorb light and are an ideal contrast agent for PAI. One of the applications of PAI in SCT is to provide guidance for accurate stem cell transplantation. PAI-guided stem cell transplantation using gold-based contrast agents has been reported [28, 56].

## 5. Selecting an Imaging Modality

It is important to select an appropriate imaging approach depending on the application, the experimental subject under study, the goal of the experiment, and so forth. Fluorescent imaging and bioluminescent imaging are preferred in the fundamental research of stem cell cultures, cell sorting in SCT, and small animal models in preclinical studies (Figure 6). Bioluminescent imaging can also characterize stem cells* in vivo* regarding cell migration and survival in small animals.

Combining MRI or CT with fluorescent or bioluminescent imaging can provide additional useful anatomical information. For preclinical studies, each modality is suitable in certain stage of the therapy: ultrasound or PAI can provide assistance during the transplantation (Figure 7); MRI is ideal for checking the lesion before delivery and performing short-term monitoring of the grafted stem cells (Figure 4); and using reporter genes in PET/CT or SPECT/CT offers long-term tracking and can provide quantitative data on cell viability (Figure 5).

## 6. Future Developments

Developing biodegradable contrast agents is one of the future development directions. Degradable contrast agents can effectively reduce the cytotoxicity and potential toxicity by facilitating the clearance of the contrast materials. A common strategy is conjugating or coating contrast agents with biodegradable materials. Examples include Gd chelates conjugated with polyester dendrimers or bovine serum albumin for MRI [123, 124], PLGA-encapsulated Lumogen Red for fluorescent imaging [125], and ^99^mTc-labeled chitosan nanoparticles for SPECT [126]. These contrast agents offer intense contrast signal as well as negligible cytotoxicity or accumulation in the liver or spleen. Using naturally occurring materials such as cellulose is another option that minimizes liver and spleen uptake [127].

Developing multimodal contrast agents is another important trend because SCT requires different imaging modalities at different stages of the treatment. One example of multimodal imaging is Cerenkov luminescence imaging (CLI) where the decay of radioisotopes can emit Cerenkov radiation and is captured by sensitive optical detectors. Thus, CLI can provide deep tissue imaging via the radionuclide as well as surface-weighted signal. This has been investigated in small animals [128, 129], but CLI for stem cell tracking is not common because CLI signal is weak compared to other detection methods and the emission wavelength (UV-Vis) is not favorable for* in vivo* measurements. Furthermore, relatively long exposure times are needed to collect a sufficiently high number of optical photons in CLI. However, CLI has been demonstrated in tracking stem cells with PET and BLI. Researchers optimized lentiviral vector (LV) transduction of murine MSCs to obtain multicistronic LVs that can express firefly luciferase for BLI and hNIS for PET and CLI [130]. CLI is useful for tracking cells because CLI allows a relatively cheap and quick acquisition of data and uses clinical radioisotopes [130].

Multimodal imaging can combine the strength of each modality for a comprehensive detection. Superficial and deep penetration imaging can be achieved by combining PAI and MRI with silica-based nanoparticles [29]. SPECT or PET uses highly sensitive labels and MRI provides excellent spatial resolution and thus using a reporter gene with radioisotopes for viability information with MRI contrast agents for highly resolved anatomic information can provide short-term and long-term cell tracking with anatomical information [131, 132]. Combining fluorescent and MRI and using conjugated fluorescent dye/magnetic particle contrast agents is another common strategy [133]. In addition, indirect labeling reporter genes have been demonstrated for multimodality imaging. For example, a tyrosinase multifunctional reporter gene was demonstrated to be sensitive in PAI, MRI, and PET [76].

Increasing the viability and differential rate of stem cell by introducing growth factors such as extracellular matrix (ECM) or scaffold is a method to strengthen stem cell viability. Implanted stem cells usually have short survival time for many reasons. Extracellular matrix or scaffolds can provide a microenvironment with structural supports and chemical stimulations, which facilitates cell activities. This has been shown both* in vitro* and* in vivo*. For example, the decellularized porcine ventricular ECM can effectively support cardiac progenitor cells by showing a strong serum-induced proliferation and a resistance to apoptosis in serum starvation [134]; the poly(ethylene glycol) diacrylate (PEGDA) hydrogel stimulates the implanted MSCs in cartilage repair and causes less pain to the patients [135]. Poly-L-lactic acid (PLLA) scaffolds can prolong the survival and increase the proliferation rate of F3 cells implanted to ablated motor cortex of the corticectomized rat [136]. Taking advantages of these extra support structures can improve SCT and increase cell viability.

Imaging plays an important role in studying the biology of cancer stem cells (CSCs) as well. It might also influence the future cancer therapy methodologies such as immunotherapy, which is a variant of stem cell imaging and SCT. CSCs might be responsible for cancer development and metastasis and are also resistant to chemotherapy [137]. Existing cellular imaging modalities are mature enough to image CSCs [138], but the focus is how to distinctively label stem-like cells from the many other nonspecific background cells.

The most common approach for CSC imaging is to target unique surface markers on the CSC surface including antibody targeting of AC133^+^ glioblastoma stem cells via PET/CT [139]. An alternative is tracing CSCs based on their function. For example, blue lasers can excite the subpopulation of autofluorescent cells in pancreatic ductal adenocarcinoma, colorectal carcinoma, hepatocellular carcinoma, and non-small-cell lung carcinoma [137]. However, surface markers depend on environment condition, vary in different CSCs, and cause conflicting data [137], and using intrinsic autofluorescent cells is limited by the low depth of penetration of optical imaging. Despite this, stem cell imaging is a powerful tool to help understand the biological behavior of different types of CSCs and investigate the influence of CSCs in tumor development and the microenvironment.

## 7. Conclusion

In this review, we described the important steps in stem cell imaging including an introduction and comparison of the imaging modalities and contrast agents as well as a vision for future developments in the field. Stem cell imaging has become an indispensable part of SCT. Using the appropriate imaging modality or multiple modalities can greatly assist the diagnostics in SCT.
